# “You’re still online…who are you talking to, love?”: the Affective-Sexual Violence Scale in young couples

**DOI:** 10.3389/fpsyg.2026.1768548

**Published:** 2026-03-25

**Authors:** Tomás Cámara-Pastor, Javier Ortuño-Sierra, Andrea Gutiérrez-García, Raquel Falcó

**Affiliations:** Department of Educational Sciences, University of La Rioja, La Rioja, Spain

**Keywords:** intimate partner, screening, self-report, validation, violence, youth

## Abstract

Violence in affective-sexual relationships constitutes a growing psychosocial challenge during adolescence and youth, exacerbated by persistent gender inequalities and the digitalization of intimacy. Despite the relevance of this phenomenon, its assessment still relies on instruments with psychometric limitations and insufficient coverage of its technological perpetuation. In response to this need, the present study developed and validated the Affective-Sexual Violence Scale (EVAS), a brief, multidimensional, psychometrically valid and reliable self-report instrument designed to comprehensively map affective-sexual violence among Spanish youth, in line with international quality standards. The study was structured in three sequential phases: (1) review of instruments and development of an item bank; (2) a pilot study aimed at refining and selecting items with the strongest conceptual and statistical support (*n* = 534); and (3) psychometric validation in a large and heterogeneous sample, examining factorial structure, internal consistency, multigroup equivalence, and victimization profiles according to sociodemographic factors (*n* = 1,142). Findings suggest that the EVAS is organized around a bifactor model, comprising a general violence factor and four specific dimensions (physical, psychological-emotional, controlling, and sexual), which makes it possible to capture the complexity of in-person and digital victimization. Its multidimensional nature facilitates the identification of differential profiles, provides both global and specific assessment, and proves to be an agile, reliable, and culturally appropriate tool for research, screening, evaluation, and monitoring of preventive programs. It is thus proposed as an innovative tool for understanding how affective-sexual violence manifests in youth and for guiding strategies to prevent it and to promote healthier relationships, with clear potential for use in educational, community, and clinical settings.

## Introduction

Adolescence is a key stage in the life course, characterized by profound biological, cognitive, and psychosocial transformations that reshape identity, autonomy, and sexuality (Copeland, 2025). This stage represents a turning point marked by exploration and the transition to adult life. In the relational domain, intimate bonds are redefined and affective experiences increase, which calls for a specific approach to adolescents’ overall health grounded in the most recent scientific evidence (Alderman et al., 2019; Baams and Kaufman, 2023). Adapting to these changes heightens vulnerability to mental health problems, especially when early detection and preventive intervention strategies are not implemented. Evidence supports the potential of universal school-based and positive development interventions, as well as validated digital approaches that enhance access to and personalization of support (Bantjes, 2022; Bryant et al., 2024; Mahon and Seekis, 2022). Complementarily, there is a call for youth-centered, transdiagnostic, and multidisciplinary preventive models capable of modifying psychopathological trajectories at an early stage (Colizzi et al., 2020; Ródenas-Perea et al., 2025).

Within this framework, teen dating violence (hereafter TDV) emerges as a major risk factor. The most recent longitudinal reviews show prospective associations between dating victimization and adverse outcomes, such as higher internalizing and externalizing symptoms, lower psychological wellbeing, increased substance use, as well as revictimization and the repetition of violent dynamics in later stages. These effects vary according to the type of violence and gender (Campo-Tena et al., 2023; Piolanti et al., 2023). In the Spanish context, TDV shows significant prevalence rates that warrant particular attention. In community samples of adolescents, including at-risk populations (i.e., co-occurrence of distal and proximal predisposing factors), lifetime victimization has been reported to range from 2.5 to 33.7%, with higher prevalence of sexual and digital victimization among girls, and higher physical victimization among boys (Oyarzún et al., 2021). In school samples, relevant prevalence rates have been observed for control and fear (20–22%), as well as patterns associated with social inequalities and sexist attitudes (Vives-Cases et al., 2021). In the digital sphere, a recent meta-analysis indicates that technology-mediated violence reaches significant levels among adolescents, with a particularly prominent controlling component (Li et al., 2023).

Early detection of this type of behavior is essential for implementing effective prevention strategies. Experimental and meta-analytic evidence confirms that prevention programs reduce TDV, especially physical violence, and improve knowledge and attitudes toward affective-sexual relationships (Lee and Wong, 2022; Piolanti and Foran, 2022). In at-risk adolescent populations, interventions also achieve reductions in perpetration and victimization when composite outcomes encompassing different types of violence are analyzed (Arrojo et al., 2024). These findings reinforce the need to develop instruments with adequate evidence of reliability and validity that allow for the identification and assessment of risk behaviors at early developmental stages. Sociocultural transformations, such as the persistence of sexism and traditional gender norms, together with the digitalization of relationships, have reshaped violent dynamics in adolescent dating. In the Spanish population, sexism predicts both perpetration and victimization, with mediating roles of empathy and assertiveness (Villanueva-Blasco et al., 2024). Complementarily, technology-mediated violence amplifies scenarios of control, monitoring, and harassment, with particularly severe consequences for girls. It is therefore essential to explicitly incorporate forms of digital violence into screening instruments (Afrouz and Vassos, 2024).

Despite advances in the field, psychometric gaps persist in instruments that assess adolescent dating violence. Methodological reviews, such as COSMIN-based ones, highlight deficits in content validity, the absence of studies on measurement error and responsiveness, and limited cross-cultural validity (Tarriño-Concejero et al., 2023). In measures related to social and gender norms linked to TDV, conceptual heterogeneity and limited comparability are observed, which hinders the synthesis of evidence (Meiksin et al., 2023). In the digital domain, inconsistencies among instruments and a limited number of validation studies have been identified, underscoring the need for updated, multidimensional, and culturally adapted tools (Rodríguez-deArriba et al., 2021). Considering the above, the prevalence and impact of TDV, together with the role of gender norms and the expansion of digital violence, confirm the need to develop instruments that integrate different dimensions and types of in-person and digital violence (Cava et al., 2023; Martínez-Soto and Ibabe, 2024). Although there are well-established instruments to assess in-person violence, their coverage of the digital modality remains uneven, which supports the relevance of integrative proposals (Rodríguez-Franco et al., 2022).

Several self-report measures are available to assess TDV, including multidimensional questionnaires validated in Spanish youth (e.g., the EMVN; García-Carpintero et al., 2018) and more recent tools that jointly address victimization and perpetration (e.g., DVQ-VP; Rodríguez-Franco et al., 2022). However, our preliminary review identified three recurring limitations for adolescent screening and research: (a) the incorporation of technology-mediated behaviors is often uneven or treated as an add-on, despite their current relevance; (b) multidimensional instruments can entail substantial respondent burden, limiting feasibility for school-based screening and prevention-program monitoring; and (c) as highlighted by COSMIN-based reviews, evidence gaps persist in key properties such as content validity and cross-cultural validity in adolescent samples (Rodríguez-deArriba et al., 2021; Tarriño-Concejero et al., 2023). These limitations justified the development of the EVAS as a brief, culturally adapted, multidimensional instrument explicitly designed to integrate in-person and digital manifestations of TDV in Spanish youth.

Consequently, the overall aim of the present study was to develop and validate a self-report instrument for the multidimensional assessment of TDV in Spanish youth: *the Affective-Sexual Violence Scale* (EVAS, acronym in Spanish). To this end, the following specific objectives were proposed: O_1_) to develop an item bank for the exploration of TDV in its in-person and digital manifestations in Spanish youth, obtaining prevalence rates; O_2_) to explore and select the items with the strongest conceptual and statistical support (based on a pilot study), proposing a preliminary factorial structure; O_3_) to provide evidences of internal structure for the EVAS; O_4_) to study the relationship of the EVAS with different indicators of psychological difficulties and emotional wellbeing; O_5_) to estimate the reliability of the EVAS scores; and O_6_) to analyze sociodemographic correlates (gender and age) in relation to TDV.

## Materials and methods

### Participants and procedure

The study followed a methodological, phase-based instrument-development and psychometric validation design implemented in three consecutive phases. Phase 1 corresponded to instrument development (methodological design), whereas Phases 2 (pilot study) and 3 (psychometric validation) used cross-sectional descriptive-observational designs. Data collection was carried out in public, private, and state-subsidized schools in the Autonomous Community of La Rioja, in group format, using digital devices, during school hours, and with an approximate duration of 20 min. The assessment protocol was previously approved by the Ethics Committee of the University of La Rioja (CEIm; Code: inf_CE_46_2023; approved on 3 October 2023), and all participants provided informed consent, which guaranteed anonymity, confidentiality, and voluntariness. The inclusion criteria for participation in the study were: having had at least one affective-sexual relationship, understood Spanish, and agreeing to participate voluntarily. Procedural aspects were structured in three consecutive phases: (1) instrument development, (2) pilot study with cognitive evaluation and initial psychometric refinement, and (3) psychometric validation in a large and heterogeneous sample.

#### Phase 1: instrument development

The first phase addressed the following objective: O_1_) to develop an item bank to assess TDV—both in-person and digital—in Spanish youth.

##### Expert panel

Content review was conducted by an expert panel composed of three professionals with complementary expertise relevant to the construct. One expert specialized in gender-based violence and adolescent dating violence, another in adolescent assessment and developmental appropriateness, and a third in psychometrics and instrument development methodology. All experts had between 11 and 15 years of professional experience. Their roles in the review process included evaluating item relevance and content appropriateness, assessing clarity and wording refinement, and reviewing methodological and structural aspects of the instrument. This multidisciplinary review ensured conceptual coverage, developmental suitability, and psychometric coherence prior to pilot testing.

##### Item generation

Items were generated by the research team based on (a) the prior literature and validated questionnaires on dating/digital/interpersonal violence and (b) theoretical frameworks on coercion, power dynamics, and youth victimization, mapping the initial pool to four dimensions (physical, psychological-emotional, controlling, and sexual violence). The initial pool was then screened to remove duplicates, simplify wording, and ensure developmental appropriateness and coverage of both in-person and technology-mediated behaviors.

##### Review and refinement procedure

Experts evaluated each item for clarity, relevance, conceptual coherence, and cultural appropriateness. Revisions were performed iteratively in two review rounds; disagreements were resolved by consensus. Items were retained when meeting predefined criteria (e.g., agreement on relevance/clarity), modified when partially meeting criteria or receiving substantive qualitative suggestions, and removed when showing low relevance/clarity or redundant content. Expert judgment followed international and national guidelines for instrument development, including COSMIN (Mokkink et al., 2018), the Standards for Educational and Psychological Testing (American Psychological Association [APA], 2014), and relevant psychometric recommendations (e.g., DeVellis, 2017; Furr and Bacharach, 2014; Muñiz and Fonseca-Pedrero, 2019), as well as national recommendations from the Consejo General de Colegios Oficiales de Psicólogos (2020). This process yielded a clear, coherent, and culturally adapted preliminary version of 55 items, suitable for pilot testing.

#### Phase 2: pilot study: cognitive evaluation and initial psychometric refinement

The second phase empirically evaluated the preliminary 55-item version to assess its comprehensibility, cultural appropriateness, and initial psychometric performance. A cross-sectional design was used with a pilot sample of adolescents and young people (*n* = 534) who met the general inclusion criteria. In addition to cognitive evaluation (face validity within content validity), Phase 2 included an EFA-based item-reduction step (Objective O2) to refine the initial pool and obtain the abbreviated 19-item version for subsequent confirmatory testing in Phase 3.

Qualitative pretesting (comprehensibility/face validity within content validity). First, comprehensibility and face validity were examined through focus groups, following international recommendations for qualitative item evaluation (Beatty and Willis, 2007; Willis, 2015). 3 focus groups were conducted (approximately 12 participants per group), guided by a topic script focused on item wording, interpretation, ambiguity, and contextual/cultural adequacy. Sessions were moderated by a member of the research team with experience in adolescent assessment, and field notes were taken. Sessions were not audio-recorded; instead, structured field notes were taken and expanded immediately afterward by the moderator and an observer. The qualitative material was organized into categories (linguistic clarity, semantic ambiguity, contextual adequacy, and cultural sensitivity) and used to introduce targeted wording refinements before quantitative testing. To ensure rigor, coding was conducted by 5 researchers and discrepancies were resolved by consensus within the team.

Quantitative pilot testing (initial psychometric refinement and item reduction). Subsequently, the revised EVAS-55 was administered in a quantitative pilot study (cross-sectional; *n* = 534) to support transparent item reduction (Objective O2). Item refinement combined distributional checks and EFA-based screening, with iterative elimination guided by prespecified criteria: (a) primary factor loadings < 0.40, (b) cross-loadings ≥ 0.30, (c) low communalities, and (d) redundancy and content coverage across the four theoretical domains. Given that the first 55-item EFA yielded low sampling adequacy (KMO = 0.50; see Results), this analysis was treated as a heuristic screening step rather than a definitive structural test, and the retained 19-item solution was subsequently subjected to stronger structural validation in Phase 3 (replicated EFA and CFA in an independent sample). Sample size (*n* = 534) was considered adequate for exploratory item-screening and reduction purposes; simulation work indicates that factor recovery depends primarily on communalities, factor overdetermination, and model complexity rather than fixed subject-to-item ratios (MacCallum et al., 1999; Mundfrom et al., 2005).

The integration of qualitative and quantitative evidence allowed for the elimination of redundant or low-performing items, resulting in an abbreviated 19-item version structured around the four theoretical dimensions. This version was considered sufficiently robust for formal evaluation in the following phase, aimed at psychometric validation with a larger and more representative sample.

#### Phase 3: psychometric validation of the EVAS

The third phase aimed to comprehensively examine the psychometric properties of the abbreviated 19-item version, assessing its internal structure, reliability, and relationship with other psychometric indicators of mental health and wellbeing in a large and heterogeneous sample of adolescents and young people. This phase constituted the empirical core of the validation process, allowing for confirmation of the conceptual and metric adequacy of the instrument after its pilot refinement.

Design and setting. Phase 3 was conducted as a cross-sectional validation study in educational centers (public, private, and state-subsidized) in the Autonomous Community of La Rioja, using a standardized group administration protocol with digital devices during school hours. The target population comprised adolescents and young people enrolled in participating educational centers. Inclusion criteria were: (a) having had at least one affective-sexual relationship, (b) understanding Spanish, and (c) voluntary participation with informed consent. Exclusion criteria were: (a) not meeting inclusion criteria and/or (b) protocols with insufficient/incomplete data for analysis. The initial validation sample included 1,142 participants (12–26 years). After excluding incomplete protocols, the final Phase 3 analytical sample comprised *n* = 941, which was subsequently split into two subsamples for EFA and CFA.

The targeted sample size for Phase 3 was set to allow a split-sample strategy (EFA/CFA in independent subsamples) and to support multigroup invariance testing across gender and age groups, while maintaining stable parameter estimation. The achieved sample size (*n* = 941; subsamples *n* = 470 and *n* = 471) provides adequate information for the planned factor-analytic and group-comparison procedures. In addition, the achieved sample and subsample sizes were considered conservative for estimating the 19-item EFA/CFA models and supporting planned invariance analyses, in line with simulation evidence indicating that SEM/CFA sample-size requirements depend on loading magnitude and model complexity (Wolf et al., 2013).

The psychometric analysis in this phase included the study of evidence related to the validity of the internal structure, the estimation of internal consistency of the EVAS scores, and the examination of validity evidence based on relationships with other relevant indicators of wellbeing and psychological indicators. Given its central role in the validation of the instrument, the technical procedures, estimators, fit criteria, and specific analytical decisions are described in detail in the section corresponding to data analysis, to maintain clarity of exposition and avoid methodological redundancies. Taken together, this phase allowed for a rigorous evaluation of the stability of the proposed theoretical model, the precision of the scores, and the coherence of the instrument with relevant psychological indicators in young populations, thus providing the empirical basis for consolidating the questionnaire as a valid and reliable measure of the phenomenon under study.

### Variables and instruments

All external instruments were administered in Phase 3 to provide validity evidence based on relationships with other variables (O4), covering (a) conceptually proximal TDV indicators for convergence (e.g., EMVN victimization subscales) and (b) indicators of psychological distress and psychosocial functioning for theoretically expected associations (e.g., SDQ, PHQ-9, GAD-7, phubbing, problematic internet use, emotion regulation, self-esteem, loneliness, mentalization, suicidal behavior, and subjective wellbeing).

#### The Affective-Sexual Violence Scale (EVAS, acronym in Spanish; developed *ad hoc*)

EVAS is a self-report instrument designed to assess affective-sexual victimization, or TDV, in dating relationships during adolescence and youth. Items are answered on a five-point Likert-type scale (1 = *Never*; 5 = *A lot*), where higher scores reflect a greater frequency of violent experiences. The scale is structured into four dimensions*: physical violence*, which encompasses behaviors aimed at causing bodily harm, such as hair pulling, slapping, or assaults with objects; *psychological-emotional violence*, which reflects verbal attacks, criticism, humiliation, or threats that affect emotional wellbeing; *controlling violence*, focused on monitoring behaviors and restrictions on autonomy and freedom to interact with other people; and *sexual violence*, which includes coercion or pressure to have sexual intercourse or perform unwanted sexual acts, as well as the dissemination of sexual material without consent. In addition to specific scores for each dimension, the EVAS allows for the calculation of a global index of *TDV*.

#### Multidimensional scale of violence in romantic relationships (Escala Multidimensional de Violencia en el Noviazgo)

The EMVN (García-Carpintero et al., 2018) is a questionnaire validated in Spanish youth populations to assess violence experienced and perpetrated in dating relationships. It consists of 64 items organized into two parallel subscales, with responses on a 6-point Likert-type scale (0 = Never; 5 = Always). In the present study, the domination and physical abuse dimensions of the victimization subscale were used as external criteria to examine the convergent validity of the EVAS. The *domination* subscale groups coercive and manipulative behaviors aimed at controlling, humiliating, or intimidating the partner, including threats of abandonment, derogatory comparisons with third parties, reproaches about the past or blame attribution, as well as behaviors intended to damage the partner’s reputation or elicit fear through verbal and emotional aggressiveness. The *physical abuse* subscale, in turn, encompasses manifestations of violence that involve direct or indirect bodily harm, threats toward the partner or close others, destruction or appropriation of belongings, mild or severe physical aggression, self-harm, and sexual coercion. Both dimensions reflect distinct forms of dating violence and provide a robust framework for externally validating the capacity of the EVAS to capture both controlling behaviors and physical and sexual aggression in young people’s affective-sexual relationships. In the present study, internal consistency for the EMVN victimization subscales was α = 0.856 and ω = 0.781 for Domination, and α = 0.786 and ω = 0.883 for Physical abuse.

#### Strengths and Difficulties Questionnaire

The SDQ (Goodman, 1997; Spanish adaptation by Ortuño-Sierra et al., 2016, 2022) is a widely used screening instrument in young populations to assess socioemotional adjustment. It consists of 25 items organized into five subscales: emotional symptoms, which reflect anxiety, sadness, or excessive worries; conduct problems, associated with disruptive or disobedient behaviors; hyperactivity/impulsivity, linked to attentional and self-regulation difficulties; peer problems, which assess conflicts, isolation, or social rejection; and prosocial behavior, which measures empathetic and cooperative tendencies. Based on these scores, the SDQ allows for the derivation of a global index of socioemotional difficulties, providing a summary profile of psychological distress. In the present study, internal consistency was α = 0.779 and ω = 0.839 for the Total Difficulties score; subscale estimates were α = 0.745 and ω = 0.817 (Emotional Symptoms), α = 0.578 and ω = 0.720 (Conduct Problems), α = 0.646 and ω = 0.725 (Hyperactivity/Inattention), α = 0.600 and ω = 0.725 (Peer Problems), and α = 0.631 and ω = 0.776 (Prosocial Behavior).

#### Patient Health Questionnaire (PHQ-9)

The PHQ-9 (Kroenke et al., 2001; Spanish adaptation by Muñoz-Navarro et al., 2017a) is a brief self-report instrument that has been widely validated for the detection of depressive symptomatology. It consists of 9 items that assess the frequency of symptoms experienced over the past 2 weeks, including depressed mood, anhedonia, sleep and appetite disturbances, fatigue, feelings of worthlessness or guilt, concentration difficulties, and suicidal ideation. Responses are given on an ordinal scale that reflects the intensity and recurrence of each symptom. The instrument yields a total score indicative of the overall level of depressive severity. In the present study, internal consistency was α = 0.881 and ω = 0.914.

#### Generalized Anxiety Disorder Scale (GAD-7)

The GAD-7 (Spitzer et al., 2006; Spanish adaptation by Muñoz-Navarro et al., 2017b) is a brief self-report instrument designed to assess the frequency and intensity of symptoms associated with generalized anxiety disorder over the past 2 weeks. Its 7 items cover persistent nervousness or tension, excessive and difficult-to-control worry, difficulty relaxing, psychomotor restlessness, irritability, and anticipatory fear that something negative may happen. Responses are recorded on a Likert-type frequency scale, yielding a total score that reflects the overall level of anxiety symptomatology. Its use is widely supported in clinical and research settings for screening, monitoring, and severity classification. In the present study, internal consistency was α = 0.894 and ω = 0.923.

#### Phubbing Scale

The PS (Barbed-Castrejón et al., 2024) assesses the frequency with which a person interrupts or impairs immediate social interaction due to mobile phone use. It refers to the act of becoming absorbed in the device and ignoring those who are physically present. It consists of 10 items with a five-point Likert response format based on behavioral frequency. In the present study, internal consistency was α = 0.776 and ω = 0.827.

#### Compulsive Internet Use Scale

The CIUS (Meerkerk et al., 2009; Spanish adaptation by Ortuño-Sierra et al., 2024) is a 14-item scale designed to assess problematic and compulsive internet use. Its items tap key dimensions of behavioral addiction, such as loss of control over time spent online, interference with daily life and interpersonal relationships, use of the internet as an emotion regulation strategy, and persistence in use despite negative consequences. Items are answered using a frequency-based response format, where higher scores indicate greater severity of compulsive use behaviors. In the present study, internal consistency was α = 0.902 and ω = 0.922.

#### Emotion Regulation Questionnaire

The ERQ (Gross and John, 2003; Spanish adaptation by Cabello et al., 2013) is a 10-item instrument designed to assess the habitual use of intrapersonal emotion regulation strategies. It comprises two differentiated subscales: cognitive reappraisal, which reflects the tendency to modify the interpretation of a situation to alter its emotional impact, and expressive suppression, which assesses the extent to which the outward expression of emotions is inhibited. Items are answered on an agreement scale, where higher scores indicate greater use of each strategy. In the present study, internal consistency was α = 0.795 and ω = 0.805 for Cognitive Reappraisal and α = 0.750 and ω = 0.753 for Expressive Suppression.

#### Interpersonal Emotion Regulation Questionnaire

The I-ERQ (Hofmann et al., 2016; Spanish adaptation by Gómez-Ortiz et al., 2016) is a 20-item questionnaire that assesses the tendency to regulate emotional states through interaction with other people. The instrument captures strategies such as seeking support or comfort, sharing emotional experiences, or relying on the presence and guidance of others to manage intense affect. Responses are given on a Likert-type scale of frequency or perceived truthfulness, where higher scores indicate greater use of interpersonal emotion regulation strategies. In the present study, internal consistency was α = 0.902 and ω = 0.918.

#### Rosenberg Self-Esteem Scale

The RSE (Rosenberg, 1965) is a classic 10-item instrument designed to assess global self-esteem, understood as the general evaluation individuals make of their own worth, self-respect, and degree of self-acceptance. The items include positive and negative statements about self-concept, which are answered on a Likert-type agreement–disagreement scale. Higher scores reflect higher levels of perceived self-esteem. In the present study, internal consistency was α = 0.874 and ω = 0.909.

#### Revised UCLA Loneliness Scale

The RULS (Russell et al., 1978; Spanish adaptation by Vázquez Morejón and Jiménez, 1994) is a 10-item self-report instrument that assesses the subjective experience of loneliness and social disconnection in adolescents and young people. The items explore feelings of isolation, lack of companionship, difficulties in establishing close relationships, and perceived lack of integration within the peer group. Responses are recorded on a Likert-type frequency scale, where higher scores indicate a greater perception of loneliness. In the present study, internal consistency was α = 0.884 and ω = 0.912.

#### Reflective Functioning Questionnaire

RFQ-8 (Fonagy et al., 2016; Spanish adaptation by Ruiz-Parra et al., 2023) is a brief questionnaire designed to assess the capacity for reflective functioning or mentalization. It consists of 8 items that explore the individual’s tendency to reflect on their own and others’ mental states (thoughts, emotions, intentions), as well as the degree of certainty or uncertainty in interpreting those states. Responses are collected on a Likert-type agreement scale, where higher scores reflect greater development of reflective functioning. In the present study, internal consistency was α = 0.784 and ω = 0.796.

#### Escala para la evaluación de la conducta suicida en adolescentes (SENTIA)

The SENTIA (Díez-Gómez et al., 2021) is a brief screening instrument designed to detect suicidal ideation and behavior in adolescents. It consists of 5 dichotomous items that assess the recent presence of thoughts of death, self-harm ideation, suicidal planning, and self-injurious behaviors or previous attempts. In the present study, internal consistency was KR-21 = 0.82.

#### Personal wellbeing index—school children

The PWI-SC (Cummins and Lau, 2005) is a questionnaire designed to assess subjective wellbeing in child and adolescent populations. It consists of 8 items that explore satisfaction across different life domains: standard of living, health, personal achievements, interpersonal relationships, safety, sense of community belonging, prospects, and overall satisfaction with life. Each item is answered using a numerical satisfaction scale, allowing for the derivation of both domain-specific scores and a global index of subjective wellbeing. In the present study, internal consistency was α = 0.947 and ω = 0.948.

In Phase 3, analyses were organized to address: O3 (internal structure: replicated EFA in subsample 1 and CFA model comparison in subsample 2, including invariance by gender and age), O4 (relationships with external measures: correlations between EVAS scores and external indicators), O5 (reliability: α and ω for subscales/total), and O6 (sociodemographic correlates: group comparisons by gender and age). Full technical specifications are detailed in the Data Analysis section.

### Data analysis

Data processing was carried out using Mplus software (version 8.7; Muthén and Muthén, 1998-2017) and JAMOVI (version 2.4.11; The Jamovi Project, 2024). The statistical procedure employed to address the specific objectives of the study is described below:

#### O_2_: selection of items with the strongest conceptual and statistical support

With the aim of refining the initial item bank and constructing a parsimonious measure of TDV, an Exploratory Factor Analysis (EFA) was conducted on the preliminary version of the questionnaire, composed of 55 items distributed across four theoretical dimensions: physical violence, psychological-emotional violence, controlling violence, and sexual violence. The analysis was performed on a pilot sample (*n* = 534), based on the Pearson correlation matrix, and its adequacy was evaluated using the Kaiser-Meyer-Olkin (KMO) index (Kaiser, 1970), Bartlett’s test of sphericity (Bartlett, 1950), parallel analysis (Horn, 1965), and scree plot inspection (Cattell, 1966). In addition, item-level Measures of Sampling Adequacy (MSA) were inspected to support screening decisions (see Supplementary Table 2). In this pilot step, Pearson correlations were used as an initial screening approach during item reduction; however, given the ordinal nature and skewness of the retained EVAS items, Phase 3 structural analyses were conducted using polychoric matrices for EFA and robust estimators for CFA, which we now clarify to improve methodological coherence. The number of factors to be retained was determined by combining theoretical and empirical criteria, using parallel analysis and scree plot inspection to contrast the proposed four-dimensional model. Factor extraction was carried out using principal axis factoring with oblimin oblique rotation, given the potential correlation between dimensions. The final selection of items was based on both statistical and content-related criteria, iteratively eliminating those with low factor loadings, high cross-loadings, low communalities, or redundant content. Specifically, items were removed when they showed primary loadings < 0.40, cross-loadings ≥ 0.30, or low communalities, and when content review indicated redundancy or insufficient conceptual representativeness. Item removal was performed iteratively, re-estimating the factor solution after each elimination step until a stable and interpretable four-factor structure was obtained. A second factor solution was then estimated using the retained items, and Cronbach’s alpha coefficients (α; Cronbach, 1951) were calculated to assess the internal consistency of each subscale. This procedure made it possible to generate hypothesized models that combine a general factor, representing the common variance, with four specific factors reflecting differentiated dimensions of the metaconstruct. For transparency, the full EVAS-55 EFA output (pattern matrix with all loadings, communalities, and MSA) is provided in Supplementary Table 2.

Subsequently, these models underwent further structural validity analyses using a larger, more representative sample (*n* = 941). The cases were randomly divided into two subsamples of similar size: *subsample 1* (*n* = 470) was used to analyze once again the internal structure of the abbreviated 19-item version of the EVAS using EFA, while subsample 2 (*n* = 471) was reserved for Confirmatory Factor Analysis (CFA). In subsample 1, the replication of EFA followed the same statistical protocol described above: polychoric correlation matrix, principal axis extraction and oblimin rotation, and Cronbach’s alpha coefficients. The number of factors was evaluated using parallel analysis, but the final decision to retain four factors was again based on consistency with the theoretical model and the interpretability of the solution. The following subsection details the CFA procedure with subsample 2. This division was carried out using a random assignment procedure generated by statistical software (approximately 50/50), resulting in subsample 1 (*n* = 470) for EFA and subsample 2 (*n* = 471) for CFA.

#### O_3_: evidences about the internal structure of the EVAS

From a conceptual standpoint, EVAS was specified as four related domains of TDV (physical, psychological-emotional, controlling, and sexual violence). Because these domains are expected to share variance due to an overarching TDV metaconstruct, we also tested a second-order hierarchical model in which a higher-order TDV factor explains the covariance among the first-order domains, to inform the defensibility of a total score. First, with the aim to assess the construct validity of the EVAS model, CFAs with subsample 2 were performed comparing five configurations derived from the structure obtained in the previous EFA: unifactorial, four correlated factors, four independent factors, second-order hierarchical, and bifactor. The goodness of fit of each model was evaluated (maximum likelihood estimator with robust standard errors; MLR) using chi-square (χ^2^), degrees of freedom (df), significance level (p), Comparative Fit Index (CFI), Tucker–Lewis Index (TLI), Root Mean Square Error of Approximation (RMSEA) with 90% confidence interval, and Standardized Root Mean Square Residual (SRMR). Because χ^2^ is highly sensitive to sample size, we report χ^2^ and its *p*-value but rely primarily on approximate fit indices. The following values were considered indicative of good fit: CFI and TLI ≥ 0.95, RMSEA ≤ 0.06, and SRMR ≤ 0.08 (Bollen, 1989; Bentler, 1990; Hu and Bentler, 1999; Marsh et al., 2004; Steiger, 1990; Tucker and Lewis, 1973).

Given that bifactor models are more parameterized and can show improved global fit due to increased flexibility, model comparisons were additionally complemented with information criteria (AIC, BIC, and sample-size adjusted BIC; Akaike, 1974; Schwarz, 1978; Sclove, 1987). To aid interpretation beyond fit, we also computed ancillary bifactor indices (e.g., ECV, PUC, ωH, and ωHS) following Rodríguez et al. (2016). Ancillary bifactor indices included Explained Common Variance (ECV), Percent of Uncontaminated Correlations (PUC), omega hierarchical (ωH), and omega hierarchical subscale (ωHS). As indicative guidelines, a dominant general factor is typically supported by high ωH (e.g., ≥ 0.80) together with higher ECV (e.g., ≥ 0.70), whereas low ωHS values suggest limited reliable subscale variance beyond the general factor.

In addition, measurement equivalence of the EVAS was evaluated as a function of the sociodemographic variables gender (male vs. female) and age (≤ 16 years vs. ≥ 17 years). Age groups were dichotomized to contrast middle adolescence with late adolescence/early adulthood, and to reflect the key educational transition in Spain, where compulsory secondary education normally ends at 16 years of age (Sawyer et al., 2018). Three levels were examined: configural, metric, and scalar. The first tests whether the items relate to the factors according to the theoretical structure; the second, whether item–factor loadings are comparable across groups; and the third, whether response thresholds are similar between groups. Measurement stability was assumed when changes in fit indices met the criteria ΔCFI/TLI ≤ 0.01 (Cheung and Rensvold, 2002) and ΔRMSEA ≤ 0.015 (Chen, 2007), thereby guaranteeing the comparability of scores.

#### O_4_: evidences of relation with other indicators of emotional wellbeing and mental health

The relationship between EVAS and other psychometric instruments of psychological problems and wellbeing were examined using total sample (*n* = 941) and Pearson correlations (*r*), interpreting their magnitude according to Cohen (1988): *r* ≈0.10 weak, *r* ≈0.30 moderate, and *r*≈0.50 strong. In this way, the CFA made it possible to evaluate the structural coherence of the models derived from the EFA, while the associations with external criteria provided evidence of conceptual convergence and divergence, thereby strengthening the construct validity of TDV. Given the ordinal response format and the skewed distributions expected for low-prevalence behaviors, distributional diagnostics (e.g., skewness/kurtosis and graphical inspection) were considered. Pearson’s r was retained as a robust estimator of association patterns in a large sample and to facilitate comparability with prior validation studies; as a sensitivity analysis, Spearman rank correlations were also examined, yielding the same direction and substantive interpretation of results.

#### O_5_: internal consistency of the scores

The internal consistency of the scores was estimated using Cronbach’s alpha (α; Cronbach, 1951) and McDonald’s omega (ω; McDonald, 1999), with values ≥ 0.70 considered indicative of optimal internal consistency (Gu et al., 2017). An item-deletion analysis was also conducted for each subscale.

#### O_6_: sociodemographic correlates of TDV

To examine the association between sociodemographic variables (gender and age) and levels of victimization on the EVAS, independent-samples *t*-tests were conducted. This analysis made it possible to compare the means of two independent groups and determine whether statistically significant differences existed. Student’s *t* statistics, degrees of freedom (*df*), *p*-values (*p*), and Cohen’s effect size (*d*) were reported, including its 95% confidence interval, in order to evaluate both the statistical significance and the practical magnitude of the differences observed between comparison groups (i.e., to identify sociodemographic profiles of victimization).

## Results

### Phase 1: instrument development

After two expert-review rounds, the initial item pool was refined for clarity, relevance, and cultural adequacy, resulting in a preliminary 55-item EVAS version for pilot testing.

### Phase 2: pilot study: item reduction (EVAS-55 to EVAS-19)

#### In search of the final set of items

##### Pilot study: EVAS-55 items (*n* = 534)

The initial EFA was conducted on the Pearson correlation matrix of the 55 items. The KMO index was 0.50, indicating low sampling adequacy and placing the solution at the lower bound of what is considered acceptable. However, Bartlett’s test of sphericity was significant [χ^2^_(1_, _485)_ ≈ 6073.19, *p* < 0.001], showing that the correlation matrix differed from the identity and contained sufficient inter-item relationships to justify a factor analysis. Parallel analysis and the scree plot supported the extraction of four factors, in line with the theoretical model distinguishing physical violence, psychological-emotional violence, controlling violence, and sexual violence. The four-factor solution, obtained using principal axis factoring with oblimin rotation, explained approximately 62.6% of the total variance. Due to the low sampling adequacy (KMO = 0.50) and the sparsity/low inter-item correlations expected for low-prevalence behaviors, several numerical issues emerged; therefore, this first solution was used primarily as a guide for item refinement, with its structure remaining subject to subsequent confirmation through EFA/CFA in an independent sample. For transparency, the detailed output of this initial EVAS-55 EFA is provided as Supplementary Table 2.

Applying statistical criteria (main loadings < 0.40, cross-loadings ≥ 0.30, low communalities) and content-related criteria, the least informative items were progressively eliminated until an abbreviated 19-item version was obtained. The resulting scale was organized into four subscales: physical violence (4 items), psychological-emotional violence (5 items), controlling violence (5 items), and sexual violence (5 items). A new correlation matrix was estimated for these 19 items, yielding an overall KMO of.84 and a significant Bartlett’s test [χ^2^_(171)_ = 2124.11, *p* < 0.001], indicating a substantial improvement in sampling adequacy. The EFA using principal axis factoring with oblimin rotation confirmed the presence of four factors, which jointly explained 77.8% of the total variance. The controlling violence items loaded on the first factor (λ between 0.82 and 0.91), the physical violence items on the second (λ between 0.62 and 0.95), the psychological-emotional violence items on the third (λ between 0.65 and 0.86), and the sexual violence items on the fourth (λ between 0.51 and 0.71). Some items showed small secondary loadings, but the overall pattern was consistent with the proposed theoretical dimensions.

In addition, the subscales of this abbreviated version showed high internal consistency in the pilot sample: α = 0.91 for physical violence, α = 0.94 for psychological-emotional violence, α = 0.96 for controlling violence, and α = 0.92 for sexual violence. These results support the reduction from 55 to 19 items and provide a solid empirical basis for subjecting the four-factor structure to confirmatory factor analysis in an independent sample.

### Phase 3: psychometric validation results

#### Main study: EVAS-19 items (subsample 1: *n* = 470)

The initial sample of main study consisted of 1,142 participants aged between 12 and 26 years. After removing protocols with incomplete data, the final sample comprised 941 participants (*M* = 16.88, *SD* = 2.61). Regarding gender, 381 participants (40.5%) identified as male, 543 (57.7%) as female, 8 (0.9%) selected “prefer not to answer,” and 3 (0.3%) indicated “other.” Sampling was carried out by convenience and was conditioned by the accessibility of educational centers and their availability to participate. The AFE replication with this subsample reaffirmed the estimates described above, suggesting a factorial structure articulated in four latent factors. The KMO index was 0.83, Bartlett’s sphericity test proved significant [χ^2^_(171)_ ≈ 13819.36, *p* < 0.001], and the explained variance ranged around 79.6%. The factor loadings (λ) ranged from 0.53 to 0.86 for the first factor (i.e., controlling violence), from 0.70 to 0.99 for the second (i.e., psychological-emotional violence), from 0.56 to 0.89 for the third (i.e., physical violence), and from 0.43 to 0.82 for the fourth (i.e., sexual violence). Internal consistency (α) showed values of 0.89, 0.86, 0.85, and 0.88, respectively.

### Mapping affective-sexual violence

Preliminarily, Table 1 presents the descriptive statistics of the EVAS-19 items, including the main indices of central tendency, dispersion, and sample distribution. Overall, the data show a positively skewed and markedly leptokurtic behavior (kurtosis > 0), which translates into a tendency to respond to the items using the lower end of the response scale (i.e., low frequency). Among the most frequent manifestations of violence in this sample are pressuring, humiliating, and controlling behaviors: receiving constant criticism about what one says or does (item 6), being insulted or verbally abused (item 7), being belittled in front of others (item 8), constant monitoring of the mobile phone and of the “last seen” status on WhatsApp (items 10 and 11), and engaging in sexual intercourse to please the partner or out of fear (item 16). By contrast, the least frequently reported behaviors correspond to more severe forms of violence, including physical assault with a weapon or dangerous object (item 3), having sexual intercourse under the influence of alcohol or drugs without being able to refuse (item 18), the non-consensual sending of sexual photos or videos (item 19), being slapped or punched (item 2), and being pulled by the hair (item 1). This pattern shows that, although episodes of extreme physical violence and sexual coercion are relatively infrequent, manifestations of psychological and controlling violence are more regular, underscoring the importance of identifying and addressing these subtle yet persistent dimensions of affective-sexual violence in adolescence.

**TABLE 1:** Response prevalence (%) and descriptive statistics for the EVAS items.

*Item	1 Never	2 Very little	3 A little	4 Quite a lot	5 A lot	*M*	*SD*	Skewness	Kurtosis
1	85.3	8.0	3.9	1.7	1.1	1.25	0.71	3.27	11.11
2	86.2	8.0	3.4	1.4	1.1	1.23	0.68	3.49	12.99
3	94.2	2.1	1.9	1.1	0.7	1.12	0.55	5.10	27.21
4	87.6	6.1	3.3	1.9	1.2	1.23	0.71	3.51	12.50
5	86.9	6.7	3.6	1.8	1.0	1.23	0.69	3.43	12.10
6	74.4	14.1	6.8	3.0	1.7	1.43	0.88	2.25	4.71
7	75.1	13.3	5.5	3.5	2.6	1.45	0.94	2.30	4.73
8	82.1	8.7	4.3	2.4	2.4	1.34	0.87	2.83	7.59
9	88.9	4.8	3.1	1.5	1.7	1.22	0.73	3.71	13.88
10	84.8	9.0	3.0	1.9	1.3	1.26	0.72	3.33	11.50
11	86.9	5.5	4.3	2.0	1.3	1.25	0.74	3.28	10.69
12	82.6	8.9	3.9	2.8	1.8	1.32	0.83	2.88	7.99
13	85.1	8.0	3.1	2.2	1.6	1.27	0.77	3.25	10.56
14	87.4	5.3	3.1	2.4	1.8	1.26	0.79	3.33	10.73
15	84.9	8.0	3.8	2.0	1.3	1.27	0.74	3.19	10.34
16	84.8	7.5	4.1	2.4	1.1	1.27	0.75	3.07	9.32
17	89.3	48	3.4	1.8	0.7	1.20	0.65	3.68	13.87
18	91.9	2.8	3.3	1.4	0.6	1.16	0.60	4.15	17.74
19	91.7	3.1	2.8	1.2	1.3	1.17	0.65	4.26	18.60

*n* = 941. Range = 1–5. *The EVAS items are available in Supplementary material.

### How is the EVAS structured? Exploring the multigroup equivalence

**FIGURE 1:**
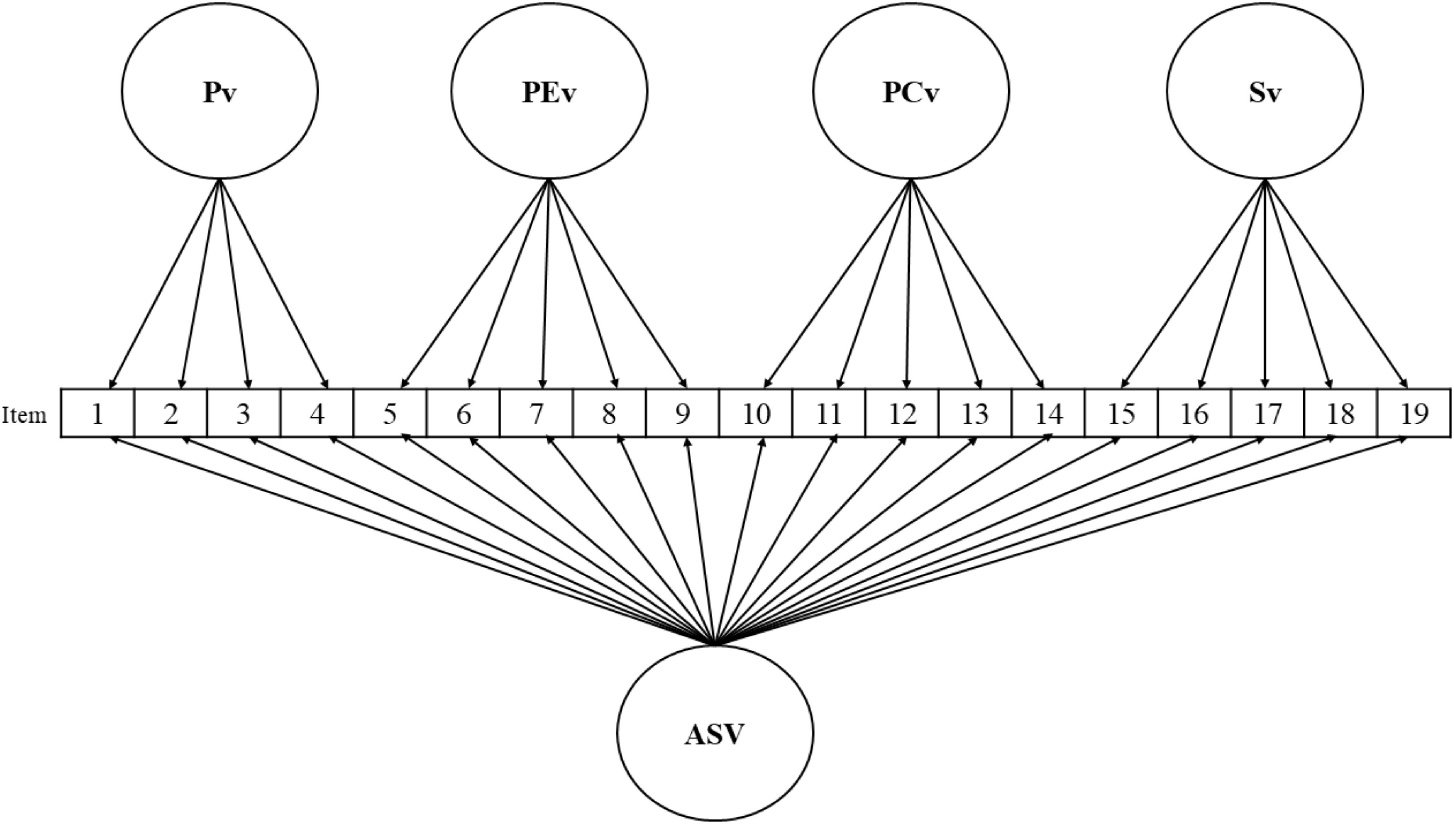
Hypothesized bifactor model. Model (e) tested in CFA. Pv, physical violence; PEv, psychological-emotional violence; PCv, psychological control violence; Sv, sexual violence; ASV, affective-sexual violence.

Regarding the analyses of structural validity, Table 2 summarizes the CFA results on subsample 2 (*n* = 471). Both the unifactorial structure (model a) and the four-independent-factor solution (model c) showed clearly insufficient fit indices, which rules out both configurations as valid representations of the latent structure of the EVAS. By contrast, the models of four correlated factors (b), second-order hierarchical (d), and bifactor (e) achieved adequate and comparable fit (CFI and TLI ≥ 0.95, RMSEA ≤ 0.06, and SRMR ≤ 0.08); χ^2^ was significant (*p* < 0.001), as expected given the large sample size. Although the differences among these three models were small in terms of overall goodness of fit, the bifactor model (e) showed the best performance, yielding the highest CFI and TLI values and the lowest RMSEA and SRMR values. This solution is also conceptually coherent, as it distinguishes a general factor that captures the variance shared by all items and four orthogonal specific factors that reflect the differentiated dimensions of the instrument. Figure 1 depicts the schematic representation of the hypothesized bifactor model, and Table 3 presents the corresponding standardized factor loadings. As can be seen, all the factor loadings were statistically significant.

**TABLE 2:** CFA of hypothesized models and multigroup measurement invariance.

Hypothesized models		χ^2^	*df*	*p*	CFI	TLI	RMSEA (90% CI)	SRMR	Δ CFI	Δ TLI	Δ RMSEA
(a) Unifactorial		753.076	152	< 0.001	0.803	0.778	0.065 (0.060, 0.069)	0.074			
(b) 4 Correlated factors		3218.528	171	< 0.001	0.952	0.943	0.033 (0.027, 0.038)	0.046			
(c) 4 Independent factors		829.983	152	< 0.001	0.778	0.750	0.069 (0.064, 0.073)	0.395			
(d) Two-level hierarchical		293.812	148	< 0.001	0.952	0.945	0.032 (0.027, 0.038)	0.046			
(e) Bifactor		197.269	127	< 0.001	0.977	0.969	0.024 (0.017, 0.031)	0.029			
Multigroup invariance (e)											
Gender	Men	240.085	133	< 0.001	0.947	0.929	0.037 (0.033,0.041)	0.032	−	−	−
	Women	175.955	127	0.003	0.981	0.974	0.026 (0.016, 0.034)	0.030	−	−	−
	1. Configural	366.120	254	< 0.001	0.965	0.952	0.031 (0.024, 0.038)	0.035	−	−	−
	2. Metric	441.846	287	< 0.001	0.955	0.942	0.034 (0.028, 0.040)	0.062	0.010	0.010	−0.003
	3. Scalar	463.991	301	< 0.001	0.949	0.942	0.034 (0.028, 0.040)	0.060	0.006	< 0.001	<0.001
Age	≤ 16 Years	185.312	133	0.002	0.970	0.962	0.028 (0.023, 0.033)	0.021	−	−	−
	≥ 17 Years	213.460	127	< 0.001	0.945	0.926	0.039 (0.030, 0.048)	0.042	−	−	−
	1. Configural	351.320	254	< 0.001	0.964	0.950	0.032 (0.025, 0.039)	0.026	−	−	−
	2. Metric	456.045	287	< 0.001	0.954	0.940	0.035 (0.029, 0.041)	0.052	0.010	0.010	−0.003
	3. Scalar	478.889	301	< 0.001	0.947	0.940	0.035 (0.029, 0.041)	0.052	0.007	< 0.001	<0.001

Total: *n* = 941, Subsample 2: *n* = 471, Men: *n* = 381, Women: *n* = 543, ≤ 16 years: *n* = 500, ≥ 17 years: *n* = 441.

**TABLE 3:** Factor loadings for the bifactor model.

Item	Pv	PEv	PCv	Sv	ASV
1	0.511	0.673	0.643	0.782	0.482
2	0.571				0.612
3	0.395				0.644
4	0.557				0.611
5					0.478
6		0.800			0.258
7		0.844			0.228
8		0.763			0.302
9		0.495			0.704
10					0.483
11			0.682		0.476
12			0.780		0.374
13			0.709		0.441
14			0.647		0.433
15					0.259
16				0.833	0.263
17				0.826	0.298
18				0.584	0.499
19				0.569	0.499

Standardized β. Pv, physical violence; PEv, psychological-emotional violence; PCv, psychological control violence; Sv, sexual violence; ASV, affective-sexual violence.

Because bifactor models are more parameterized and can show improved global fit due to increased flexibility, we complemented the comparison of competing models with information criteria. Information criteria clearly favored the bifactor solution over the second-order and correlated four-factor models (bifactor: AIC = 30000.24, BIC = 30378.72, SABIC = 30137.33; second-order: AIC = 30323.30, BIC = 30627.08, SABIC = 30433.33; four correlated factors: AIC = 30325.83, BIC = 30639.58, SABIC = 30439.48). In addition, ancillary bifactor indices (Rodríguez et al., 2016) supported a strong general TDV factor (ECV_G = 0.737; PUC = 0.789; ω = 0.969; ωH = 0.897). At the domain level, omega hierarchical subscale coefficients suggested comparatively modest residual reliability for most specific factors (ωHS = 0.268 for Physical, 0.128 for Psychological-emotional, and 0.153 for Controlling), whereas the Sexual domain retained comparatively higher specificity (ωHS = 0.378), indicating that EVAS scores can be meaningfully interpreted as a total TDV score while some domains—particularly Sexual—may retain incremental domain-specific variance depending on the intended use.

The results of the factorial invariance analysis for the bifactor model confirmed multigroup equivalence, that is, similarity in measurement by gender (men vs. women) and age group (16 years or younger vs. 17 years or older). The indicators of configural, metric, and scalar invariance met the established criteria—i.e., ΔCFI/TLI ≤ 0.01 and ΔRMSEA ≤ 0.015—and the model fit indices were optimal in all comparison groups, indicating that the EVAS maintains its structure and psychometric functioning consistently across these subpopulations.

### EVAS vs. other psychometric indicators

The TDV subscales showed positive, significant, and moderate-to-high correlations with conceptually related dimensions of the EMVN, such as dominance and physical abuse, supporting the convergence of the instrument with theoretically related constructs (see Table 4). In turn, associations with indicators of psychological distress, depressive symptomatology, anxiety, and behavioral difficulties were generally low to moderate, although statistically significant in several cases, demonstrating the capacity of the EVAS to distinguish dating victimization from other manifestations of psycho(patho)logy. Complementarily, negative and significant correlations with adaptive variables such as subjective wellbeing, self-esteem, and interpersonal emotion regulation reinforced discriminant validity: higher scores in affective-sexual violence were associated with lower levels of psychosocial adjustment and greater difficulties in socioemotional competencies. In terms of magnitude, the highest coefficients were observed between the physical, psychological-emotional, and controlling violence subscales and dominance and physical abuse, whereas associations with indicators of adaptive and problematic functioning remained in the low-to-moderate range, consistent with the differentiated nature of these constructs.

**TABLE 4:** Relationship with other psychometric indicators.

(Sub)scales		1	2	3	4	5
EVAS	1. Physical violence	1	1	1	1	1
	2. Psychological-emotional violence	0.660***				
	3. Controlling psychological violence	0.662***	0.795***			
	4. Sexual violence	0.566***	0.650***	0.664***		
	5. Affective-sexual violence (total score)	0.803***	0.911***	0.912***	0.831***	
EMVN	Dominance	0.522***	0.686***	0.697***	0.627***	0.739***
	Physical abuse	0.642***	0.708***	0.710***	0.639***	0.779***
SDQ	Psychological problems (total score)	0.182***	0.255***	0.202***	0.204***	0.246***
	Emotional problems	0.049	0.142***	0.090**	0.134***	0.124***
	Conduct problems	0.227***	0.223***	0.200***	0.155***	0.230***
	Inattention and hyperactivity	0.093**	0.157***	0.120***	0.120***	0.144***
	Interpersonal problems	−0.182***	−0.117***	−0.106***	−0.091**	−0.137***
	Prosocial behavior	0.220***	0.293***	0.253***	0.267***	0.301***
PHQ-9	Depression	0.192***	0.270***	0.226***	0.233***	0.269***
GAD-7	Generalized anxiety	0.027	0.090**	0.084**	0.056	0.079*
PS	Phubbing	0.154***	0.191***	0.197***	0.167***	0.206***
CIUS	Problematic internet use	0.025	−0.005	0.015	0.010	0.011
ERQ	Cognitive reappraisal	0.067*	0.070*	0.044	0.040	0.063*
	Expressive suppression	0.009	−0.013	0.010	0.010	0.003
I-ERQ	Interpersonal emotion regulation	−0.051	−0.132***	−0.069*	−0.120***	−0.111***
RSE	Self-esteem	0.036	0.147***	0.083**	0.118***	0.117***
RULS	Perceived loneliness	0.106***	0.120***	0.083**	0.093**	0.115***
RFQ-8	Mentalization	0.196***	0.232***	0.226***	0.226***	0.255***
SENTIA	Suicidal behavior	−0.143***	−0.164***	−0.134***	−0.196***	−0.183***
PWI-SC	Subjective wellbeing	0.182***	0.255***	0.202***	0.204***	0.246***

Significance level: *p < 0.05, **p < 0.01, ***p < 0.001.

### Internal consistency of the EVAS scores

Table 5 shows Cronbach’s alpha (α) and McDonald’s omega (ω) internal consistency coefficients for each subscale, including the analysis of individual item deletion. In the total sample, values ranged from acceptable to excellent across all dimensions, indicating solid internal reliability.

**TABLE 5:** Internal consistency of the EVAS subscales.

Subscales and items	Total		Men		Women		≤16 years		≥17 years	
	α	ω	α	ω	α	ω	α	ω	α	ω
*Pv*	0.855	0.859	0.871	0.873	0.816	0.829	0.878	0.881	0.787	0.798
If item 1 is deleted	0.834	0.839	0.828	0.832	0.814	0.824	0.858	0.862	0.757	0.768
If item 2 is deleted	0.786	0.796	0.808	0.813	0.745	0.779	0.815	0.822	0.705	0.729
If item 3 is deleted	0.833	0.837	0.860	0.864	0.788	0.798	0.861	0.864	0.758	0.768
If item 4 is deleted	0.805	0.813	0.837	0.843	0.719	0.745	0.837	0.843	0.716	0.731
*PEv*	0.902	0.905	0.891	0.893	0.907	0.912	0.915	0.916	0.891	0.898
If item 5 is deleted	0.881	0.884	0.866	0.869	0.890	0.893	0.895	0.897	0.871	0.877
If item 6 is deleted	0.873	0.879	0.876	0.879	0.868	0.876	0.900	0.902	0.846	0.858
If item 7 is deleted	0.873	0.878	0.850	0.854	0.881	0.889	0.897	0.899	0.851	0.863
If item 8 is deleted	0.875	0.882	0.860	0.865	0.884	0.893	0.886	0.890	0.868	0.880
If item 9 is deleted	0.895	0.899	0.880	0.883	0.905	0.910	0.899	0.902	0.892	0.898
*PCv*	0.910	0.912	0.893	0.893	0.913	0.916	0.920	0.921	0.900	0.902
If item 10 is deleted	0.893	0.894	0.877	0.878	0.892	0.895	0.904	0.905	0.880	0.883
If item 11 is deleted	0.888	0.889	0.862	0.863	0.892	0.896	0.898	0.899	0.876	0.879
If item 12 is deleted	0.885	0.887	0.863	0.864	0.888	0.893	0.899	0.901	0.870	0.872
If item 13 is deleted	0.884	0.886	0.859	0.861	0.887	0.893	0.901	0.902	0.865	0.869
If item 14 is deleted	0.902	0.903	0.884	0.885	0.905	0.908	0.907	0.908	0.898	0.899
*Sv*	0.899	0.901	0.910	0.913	0.883	0.888	0.932	0.934	0.861	0.865
If item 15 is deleted	0.879	0.882	0.910	0.913	0.845	0.854	0.925	0.926	0.826	0.832
If item 16 is deleted	0.865	0.868	0.880	0.886	0.842	0.851	0.913	0.917	0.800	0.807
If item 17 is deleted	0.861	0.864	0.888	0.890	0.828	0.836	0.908	0.909	0.808	0.815
If item 18 is deleted	0.887	0.891	0.888	0.892	0.880	0.885	0.913	0.916	0.861	0.867
If item 19 is deleted	0.890	0.893	0.885	0.890	0.884	0.890	0.925	0.927	0.854	0.861

α = Cronbach’s alpha, ω = McDonald’s omega. Total: *n* = 941, Men: *n* = 381, Women: *n* = 543, ≤ 16 years: *n* = 500, ≥ 17 years: *n* = 441. Pv, physical violence; PEv, psychological-emotional violence; PCv, psychological control violence; Sv, sexual violence.

When comparing by sex and age, some differences were observed: men showed slightly higher coefficients for physical and sexual violence, whereas women reported higher values for psychological-emotional and controlling violence. Similarly, participants aged 17 years or older showed slightly higher internal consistency in most subscales than those aged 16 years or younger. Deleting individual items did not substantially improve the coefficients, indicating that all items contribute consistently to their respective subscales.

### What affective-sexual victimization profiles are observed by gender and age?

Table 6 presents the results of between-group comparisons using independent samples *t*-tests. Regarding gender, statistically significant differences were observed only in physical violence, with higher scores among boys; however, the effect size was small, indicating that the magnitude of the difference is limited in practical terms. For the remaining dimensions, no significant differences were found (*p* > 0.05), reflecting a relatively homogeneous pattern between boys and girls. With respect to age, significant differences were identified in physical and sexual violence, with higher scores in the ≤ 16-year-old group, although effect sizes were likewise small. The other subscales showed stability across age groups, suggesting that manifestations of interpersonal violence follow a consistent pattern throughout adolescence.

**TABLE 6:** Descriptive statistics and intergroup comparisons by (sub)factor.

Sample	Statistic	Pv	PEv	PCv	Sv	ASV
Descriptives						
Total	*M*	1.21	1.34	1.27	1.22	1.26
	*SD*	0.31	0.49	0.43	0.33	0.30
Women	*M*	1.12	1.32	1.24	1.22	1.23
	*SD*	0.40	0.69	0.64	0.562	0.51
Men	*M*	1.30	1.35	1.29	1.19	1.28
	*SD*	0.66	0.69	0.65	0.55	0.55
≤ 16	*M*	1.26	1.30	1.26	1.18	1.25
	*SD*	0.64	0.68	0.92	0.58	0.56
≥ 17	*M*	1.15	1.38	1.29	1.26	1.27
	*SD*	0.17	0.51	0.44	0.32	0.27
Mean difference						
	*t_(*df*)_*	4.950_(922)_	0.674_(922)_	1.132_(922)_	−0.726_(922)_	1.618_(922)_
	*p*	< 0.001	0.501	0.258	0.468	0.106
	*d*	0.33	0.05	0.08	−0.05	0.11
	CI 95%	0.20, 0.46	−0.09, 0.18	−0.06, 0.21	−0.18, 0.08	−0.02, 0.24
Years	*t_(*df*)_*	3.010_(939)_	−1.651_(939)_	−0.709_(939)_	−2.057_(939)_	−0.529_(939)_
	*p*	0.003	0.099	0.479	0.040	0.597
	*d*	0.20	−0.11	−0.04	−0.13	−0.03
	CI 95%	0.07, 0.32	−0.24,0.02	−0.17, 0.08	−0.26, −0.01	−0.16, 0.09

Range = 1–5. t = Student’s t statistic; d = Cohen’s d effect size. Effect sizes were interpreted as small (≈ 0.20), medium (≈ 0.50), and large (≈ 0.80). Total: *n* = 941, Men: *n* = 381, Women: *n* = 543, ≤ 16 years: *n* = 500, ≥ 17 years: *n* = 441. Pv, physical violence; PEv, psychological-emotional violence; PCv, psychological control violence; Sv, sexual violence; ASV, affective-sexual violence.

## Discussion

The main objective of the present study was to develop and validate a brief self-report instrument for the multidimensional assessment of violence in affective-sexual relationships among Spanish adolescents and young people (Rodríguez-deArriba et al., 2021; Tarriño-Concejero et al., 2023). The results obtained in both the pilot study and the validation phase indicate that the EVAS is a psychometrically sound instrument, capable of capturing a general factor of violence in intimate relationships alongside four specific dimensions—physical, psychological-emotional, controlling, and sexual violence—with adequate reliability and validity indices, as well as equivalent measurement properties across gender and age (García-Carpintero et al., 2018; Meiksin et al., 2023). Given the exploration and instrument-development nature of the study, the specific objectives related to the construction of the item bank (O_1_) and the derivation of a preliminary factorial structure (O_2_) have been addressed previously; therefore, this section primarily integrates the implications of the findings associated with the objectives of structural validation, reliability, invariance, and sociodemographic correlates.

A methodological consideration concerns the first pilot EFA conducted on the 55-item pool, which yielded a low KMO value (0.50). In the context of TDV assessment, low endorsement rates and highly skewed item distributions are common, which can attenuate inter-item correlations and reduce sampling adequacy, even when Bartlett’s test is significant. For this reason, the initial 55-item EFA was interpreted as an item-screening procedure to support reduction and content refinement rather than as a definitive test of dimensionality. Importantly, the retained 19-item solution showed substantially improved sampling adequacy in the pilot (KMO = 0.84) and was subsequently subjected to stronger structural evaluation in Phase 3 through replicated EFA and CFA in independent subsamples, supporting the stability of the proposed structure.

Overall, the design phase and pilot study highlighted the lack of brief validated instruments that jointly integrate the different manifestations of dating violence, including those perpetrated through information and communication technologies (e.g., insistent calls, controlling messages, pressure to respond immediately, or monitoring of social networks) in Spanish youth populations (see Afrouz and Vassos, 2024; Cava et al., 2023; Li et al., 2023; Rodríguez-deArriba et al., 2021; Tarriño-Concejero et al., 2023). The combination of systematic review, expert judgment, and empirical item refinement made it possible to generate a 19-item version that is conceptually clear, adapted to the Spanish sociocultural context, and structured into four preliminary factors, which constituted the basis for subsequent extensive validation (Rodríguez-deArriba et al., 2021; Tarriño-Concejero et al., 2023).

With regard to the objective of structural validity (O_3_), the EFA and CFAs indicated that models incorporating a general factor of violence in intimate relationships showed superior fit compared with unidimensional models or models with four correlated factors. In particular, the bifactor model—with a general factor of affective-sexual violence and four orthogonal specific factors—offered an optimal balance between parsimony and fit. These findings support the conceptualization of a global construct of dating violence that coexists with specific dimensions of physical, psychological-emotional, controlling, and sexual violence (Gracia-Leiva et al., 2019). From an applied perspective, this supports the simultaneous use of a total score and subscale scores to describe differentiated victimization profiles and guide preventive and clinical interventions (Campo-Tena et al., 2023; Piolanti et al., 2023). In addition, the study of factorial invariance analyses demonstrated that the structure of the EVAS is equivalent between boys and girls and across the age ranges considered, at least at the configural, metric, and scalar levels, which allows mean differences to be interpreted as reflecting substantive variations rather than mere measurement artifacts (Meiksin et al., 2023).

Moreover, the EVAS scores were correlated with other psychometric indicators of mental health and wellbeing (O_4_). The different dimensions showed moderate correlations with the domination and physical abuse subscales of the EMVN, indicating a shared conceptual core without excessive redundancy. Physical violence was mainly associated with physical abuse, whereas psychological-emotional and controlling violence were linked to domination, reinforcing the conceptual differentiation between dimensions and aligning with previous studies that underscore the need to jointly assess different manifestations of violence in order to capture their long-term impact (Campo-Tena et al., 2023; Gracia-Leiva et al., 2019; Meiksin et al., 2023). Moreover, EVAS’s scores were consistently related (positive correlation) to indicators of psychological distress, including depressive and anxiety symptomatology, socioemotional difficulties, perceived loneliness, and suicidal ideation, which supports convergent validity by reflecting the expected connection between affective-sexual victimization and emotional consequences. These results reveal that violence in the context of intimate relationships has a relevant impact on mental health, highlighting the relevance of preventing these behaviors at early stages (Campo-Tena et al., 2023; Piolanti et al., 2023).

In turn, evidences of divergent validity were evidenced through low or inverse correlations with conceptually distinct factors associated with wellbeing and psychological resources, such as self-esteem, intrapersonal and interpersonal emotion regulation, reflective functioning, and life satisfaction. This pattern confirms that the EVAS captures a specific risk construct linked to dating violence, differentiated from positive mental health domains, and highlights the importance of assessing affective-sexual victimization independently while incorporating both risk and protective factors in clinical and preventive interpretation (Campo-Tena et al., 2023; Piolanti et al., 2023).

With regards to the internal consistency of the scores (O_5_), the EVAS showed high internal consistency coefficients for both the subscales and the total score, in line with COSMIN recommendations. The existence of a robust general factor reinforces the usefulness of the total score as an indicator of the overall severity of affective-sexual violence. From a practical scoring perspective, the low ωHS values indicate that the EVAS total score should be prioritized as the primary score for screening and monitoring purposes. Subscale scores should be interpreted with caution as they may contain limited reliable variance beyond the general factor, although the Sexual domain appears comparatively more defensible.

In sum, the inclusion of sociodemographic variables made it possible to examine the distribution of violence beyond the overall description (O_6_). In line with the literature, significant differences by gender and age were identified in several dimensions, particularly in physical and sexual violence, although with small-to-moderate effect sizes (Oyarzún et al., 2021; Villanueva-Blasco et al., 2024; Vives-Cases et al., 2021). This pattern suggests that TDV is a cross-cutting phenomenon in adolescence and youth, modulated but not determined by these variables (Gracia-Leiva et al., 2019). A relevant finding was that boys reported higher levels of physical violence victimization than girls. Although this result may seem counterintuitive from the perspective of gender-based violence, it is consistent with previous studies showing that, when low- or moderate-intensity physical acts are considered, boys may report equal or higher rates of victimization, whereas girls show greater sexual victimization and a more pronounced emotional impact (Archer, 2000; López-Barranco et al., 2022; Rubio-Garay et al., 2017). In adolescent contexts, dating violence is often bidirectional, and certain mild forms of aggression perpetrated by girls may be perceived by boys as less serious (Cuadrado-Gordillo et al., 2023; Gracia-Leiva et al., 2019).

This result can also be explained by several factors. First, many instruments, including the EVAS, record the frequency of physical acts without weighting severity, context (e.g., self-defense), or consequences (injuries, fear, control), which favors apparent “gender symmetries” (Archer, 2000; López-Barranco et al., 2022). Second, gender socialization may shape both the expression of violence and the willingness to acknowledge it; boys may report certain physical episodes with less threat to their self-image, whereas girls tend to report more clearly sexual and controlling violence and the associated fear (Cuadrado-Gordillo et al., 2023; Rubio-Garay et al., 2017; Vives-Cases et al., 2021). Third, the cross-sectional and anonymous nature of the design may facilitate boys’ disclosure of experiences that, although not labeled as “gender-based violence” in public discourse, nevertheless represent forms of victimization within intimate relationships. In any case, the accumulated evidence indicates that, even when boys report higher rates of physical acts, the most serious consequences—injuries, fear, impact on health, and continuation of the relationship—are concentrated among girls (Archer, 2000; Campo-Tena et al., 2023; Piolanti et al., 2023). Therefore, these data should be interpreted with caution: they reflect the presence of low- or moderate-intensity physical violence among boys, underscoring the need to make this dimension visible, without contradicting the evidence on the structural nature of gender-based violence or the greater overall impact on girls. Nonetheless, they highlight the need for further research on the nature, severity, and context of physical violence in young people’s relationships, incorporating indicators of harm, fear, and control, and explicitly addressing male victimization.

Taken together, these results show that the EVAS reliably and differentially captures violence in intimate relationships, distinguishing it from other related psychological processes, which positions it as a robust instrument for the comprehensive assessment of affective-sexual victimization in adolescents and young people.

### Limitations and future directions

Despite the psychometric robustness of the results, the present study has several limitations that should be considered when interpreting its findings. First, in both the pilot phase and the validation study, convenience sampling was used, conditioned by the accessibility of educational centers, which restricts population representativeness and calls for caution when generalizing the results to other geographical and sociocultural contexts. Second, the cross-sectional design of the study and the exclusive reliance on self-reports of victimization preclude the establishment of causal relationships and do not allow for the assessment of the temporal stability or sensitivity to change of the instrument. Likewise, the use of a single source of information may introduce memory biases, under- or overestimation of behaviors, and social desirability, especially in a sensitive phenomenon such as dating violence. Finally, in line with COSMIN guidelines and recent literature on the evaluation of psychological instruments, future research is recommended to examine the test–retest reliability, measurement error, responsiveness, and predictive validity of the EVAS, additionally incorporating longitudinal designs and multiple informants (for example, partners, observers, or administrative records), with the aim of obtaining more robust and contextualized evidence regarding its applicability and stability (Arrojo et al., 2024; Campo-Tena et al., 2023; Lee and Wong, 2022; Piolanti et al., 2023; Piolanti and Foran, 2022; Tarriño-Concejero et al., 2023). Additionally, content validity in Phase 1 was evaluated qualitatively through expert review and qualitative pretesting, but no quantitative content-validity indices were computed. Although COSMIN prioritizes qualitative appraisal for this property, future studies could complement expert judgment with quantitative indices to increase transparency and traceability of item-level decisions.

### Strengths and practical implications

In contrast, the present study also has several strengths that should be highlighted. First, the EVAS was developed explicitly in accordance with the quality criteria established by COSMIN, incorporating systematic procedures of content review, expert judgment, and advanced analyses of factorial structure, invariance, and reliability (Rodríguez-deArriba et al., 2021; Tarriño-Concejero et al., 2023). Second, it is a brief instrument (19 items), easy and quick to administer, and suitable for school and community settings, without representing a significant burden for adolescents and young people. Third, the scale is specifically adapted to the Spanish sociocultural context and integrates forms of violence mediated by mobile phones and information and communication technologies, faithfully reflecting current dynamics of affective-sexual relationships at this developmental stage (Afrouz and Vassos, 2024; Cava et al., 2023; Li et al., 2023; Rodríguez-deArriba et al., 2021). Finally, the combination of a general factor with four specific factors offers a flexible structure that allows both a global assessment of victimization and the identification of differentiated risk profiles (Gracia-Leiva et al., 2019).

From an applied perspective, the EVAS constitutes an appropriate early screening tool for identifying young people at risk of dating violence, with potential application in educational centers, youth services, and mental health resources, thereby facilitating referral and early intervention. The total score and the subscales make it possible to detect both situations of generalized violence and specific patterns (for example, a predominance of controlling or sexual violence), which guides the adaptation of preventive programs to the concrete needs of each group. Likewise, the psychometric robustness of the scale and its alignment with international standards position as a valid and reliable measure for outcome evaluation in preventive interventions and for the design of public policies aimed at reducing gender-based violence among young people (Arrojo et al., 2024; Lee and Wong, 2022; Piolanti and Foran, 2022).

## Conclusion

The EVAS is presented as a brief, multidimensional, and psychometrically sound self-report instrument designed to assess violence in affective-sexual relationships among Spanish adolescents and young people. The bifactor model, composed of a general factor and four specific factors (physical, psychological-emotional, controlling, and sexual violence), provides a coherent and well-fitting structure, supported by evidence of structural and convergent validity, as well as by high reliability and measurement invariance by gender and age. The integration of in-person manifestations and those mediated by mobile phones and digital technologies enhances the relevance of the instrument in a context marked by the digitalization of intimate relationships and by gender inequalities, which shape new risk scenarios. In line with COSMIN quality criteria, the findings suggest that the EVAS constitutes a benchmark tool for the early detection and monitoring of dating violence, with applications in educational settings and youth services, thereby contributing to the promotion of healthier affective-sexual relationships and to the reduction of the impact of violence during this critical developmental stage.

## Data Availability

The datasets presented in this article are not readily available because they contain sensitive information collected from adolescents and young adults about affective-sexual/dating violence. To protect participants’ confidentiality and privacy, and because explicit consent for data sharing/dissemination (including via repositories or upon request) was not obtained, the raw/anonymized dataset cannot be made publicly available or shared on request. Only aggregated results are reported in the manuscript and Supplementary material. Any further inquiries can be directed to the corresponding author.
