# Supplementary material for: “You’re still online…who are you talking to, love?”: the Affective-Sexual Violence Scale in young couples
Source: Front Psychol. 2026 Mar 25;17:1768548. doi: 10.3389/fpsyg.2026.1768548 (PMC13057489; doi:10.3389/fpsyg.2026.1768548)
Supplement: Supplementary file 1 [file Table_1.docx]

**Escala de Violencia Afectivo-Sexual, EVAS (Cámara-Pastor et al., 2026)**

| Piensa en todas las personas con las que hayas tenido una relación de pareja entendida como un vínculo afectivo con trato amoroso (no es necesario que haya estado formalmente etiquetado como “novio”, “novia” u otra denominación).  Responde a cada afirmación indicando en qué medida refleja tu experiencia, asegurándote de que no ocurrió en un contexto lúdico o de broma. | | | | | | | | | |
| --- | --- | --- | --- | --- | --- | --- | --- | --- | --- |
|  | | | | | | | | | |
| 1 | 2 | 3 | 4 | 5 | | | | | |
| Nunca  [Never] | Muy poco  [Very little] | Poco  [A little] | Bastante  [Quite a lot] | Mucho  [A lot] | | | | | |
|  | | | | | | | | | |
| Ítems | | | | | | | | | |
| **1. Te ha tirado del pelo.**  [1. Has pulled your hair.] | | | | | 1 | 2 | 3 | 4 | 5 |
| **2. Te he dado una bofetada/puñetazo.**  [2. Has slapped or punched you.] | | | | | 1 | 2 | 3 | 4 | 5 |
| **3. Te ha agredido físicamente con un cuchillo o algo similar.**  [3. Has physically assaulted you with a knife or something similar.] | | | | | 1 | 2 | 3 | 4 | 5 |
| **4. Intencionalmente, te ha tirado algún objeto con el que pudiera hacerte daño.**  [4. Has intentionally thrown an object at you that could hurt you.] | | | | | 1 | 2 | 3 | 4 | 5 |
| **5. Te ha dicho que no vales nada, algo así como que eres un fracaso de persona.**  [5. Has told you that you are worthless, or that you are a failure as a person.] | | | | | 1 | 2 | 3 | 4 | 5 |
| **6. Te critica por lo que dices o haces.**  [6. Criticizes you for what you say or do.] | | | | | 1 | 2 | 3 | 4 | 5 |
| **7. Te ha insultado o te ha hecho sentirte mal contigo misma/o.**  [7. Has insulted you or made you feel bad about yourself.] | | | | | 1 | 2 | 3 | 4 | 5 |
| **8. Te ha menospreciado o humillado delante de otras personas.**  [8. Has belittled or humiliated you in front of other people.] | | | | | 1 | 2 | 3 | 4 | 5 |
| **9. Te ha amenazado verbalmente con hacerte daño.**  [9. Has verbally threatened to harm you.] | | | | | 1 | 2 | 3 | 4 | 5 |
| **10. Te revisa el móvil para saber todo lo que haces.**  [10. Checks your mobile phone to know everything you do.] | | | | | 1 | 2 | 3 | 4 | 5 |
| **11. Revisa compulsivamente la última conexión a WhatsApp para controlarte.**  [11. Compulsively checks your last WhatsApp connection in order to control you.] | | | | | 1 | 2 | 3 | 4 | 5 |
| **12. Comprueba que lo que le cuentas es cierto a través de terceras personas porque no se fía.**  [12. Checks, through third persons, that what you tell them is true because they do not trust you.] | | | | | 1 | 2 | 3 | 4 | 5 |
| **13. Trata o ha tratado de impedir que veas a tus amistades.**  [13. Tries or has tried to prevent you from seeing your friends.] | | | | | 1 | 2 | 3 | 4 | 5 |
| **14. Te ha amenazado con dejarte si no accedías a hacer algo.**  [14. Has threatened to break up with you if you did not agree to do something.] | | | | | 1 | 2 | 3 | 4 | 5 |
| **15. Te ha insistido en realizar algún acto de tipo sexual, pese a tu negativa.**  [15. Has insisted on engaging in some kind of sexual activity despite your refusal.] | | | | | 1 | 2 | 3 | 4 | 5 |
| **16. Has mantenido relaciones sexuales para agradar o por miedo.**  [16. You have had sexual intercourse to please them or out of fear.] | | | | | 1 | 2 | 3 | 4 | 5 |
| **17. Te ha hecho hacer cosas durante la relación sexual, a pesar de decir que no querías o no te apetecía.** [17. Has made you do things during sexual intercourse even though you said that you did not want to or did not feel like it.] | | | | | 1 | 2 | 3 | 4 | 5 |
| **18. Te ha hecho mantener relaciones sexuales cuando eras incapaz de rechazarlas, debido a que estabas bajo la influencia del alcohol o las drogas.** [18. Has made you have sexual intercourse when you were unable to refuse because you were under the influence of alcohol or drugs.] | | | | | 1 | 2 | 3 | 4 | 5 |
| **19. Te ha enviado fotos o videos con contenido sexual sin que tu quisieras que te han causado malestar.** [19. Has sent you photos or videos with sexual content, against your wishes, that made you feel distressed.] | | | | | 1 | 2 | 3 | 4 | 5 |
