# Supplementary material for: “You’re still online…who are you talking to, love?”: the Affective-Sexual Violence Scale in young couples
Source: Front Psychol. 2026 Mar 25;17:1768548. doi: 10.3389/fpsyg.2026.1768548 (PMC13057489; doi:10.3389/fpsyg.2026.1768548)
Supplement: Supplementary file 2 [file Table_2.docx]

**Supplementary Table S2**

N = 534; Correlation matrix: Pearson; Extraction: Principal Axis Factoring; Rotation: Oblimin. KMO (overall) = .50; Bartlett’s test of sphericity: χ²(1485) = 6073.19, p < .001. Four-factor solution retained (parallel analysis/scree), explaining 62.6% of the total variance.

| **Item** | **F1** | **F2** | **F3** | **F4** | **h2** | **MSA** |
| --- | --- | --- | --- | --- | --- | --- |
| Item01 | 0.756 | -0.126 | 0.144 | 0.000 | 0.609 | 0.831 |
| Item02 | 0.641 | -0.372 | -0.193 | -0.146 | 0.608 | 0.543 |
| Item03 | 0.754 | 0.013 | -0.044 | 0.026 | 0.571 | 0.250 |
| Item04 | 0.727 | -0.020 | -0.076 | -0.078 | 0.541 | 0.266 |
| Item05 | 0.481 | -0.060 | 0.148 | -0.176 | 0.287 | 0.371 |
| Item06 | 0.784 | -0.192 | -0.094 | -0.095 | 0.669 | 0.496 |
| Item07 | -0.311 | -0.064 | -0.160 | 0.065 | 0.130 | 0.250 |
| Item08 | 0.530 | -0.036 | -0.017 | -0.021 | 0.283 | 0.536 |
| Item09 | 0.231 | 0.158 | -0.014 | 0.065 | 0.083 | 0.600 |
| Item10 | 0.284 | 0.060 | -0.009 | 0.076 | 0.090 | 0.598 |
| Item11 | 0.532 | 0.356 | 0.076 | 0.149 | 0.438 | 0.772 |
| Item12 | 0.539 | 0.036 | -0.277 | -0.050 | 0.371 | 0.850 |
| Item13 | 0.610 | 0.019 | 0.056 | 0.062 | 0.379 | 0.250 |
| Item14 | 0.253 | 0.004 | 0.025 | 0.136 | 0.083 | 0.628 |
| Item15 | -0.105 | 0.613 | 0.005 | 0.032 | 0.388 | 0.250 |
| Item16 | -0.323 | 0.731 | -0.032 | -0.232 | 0.693 | 0.250 |
| Item17 | -0.055 | 0.763 | -0.014 | -0.075 | 0.590 | 0.634 |
| Item18 | 0.024 | 0.486 | 0.107 | -0.220 | 0.297 | 0.250 |
| Item19 | 0.013 | 0.841 | -0.145 | 0.039 | 0.730 | 0.693 |
| Item20 | 0.123 | 0.633 | -0.085 | -0.071 | 0.428 | 0.542 |
| Item21 | 0.008 | 0.746 | 0.026 | 0.365 | 0.691 | 0.250 |
| Item22 | -0.086 | 0.454 | -0.015 | 0.062 | 0.218 | 0.611 |
| Item23 | 0.198 | 0.561 | -0.149 | 0.078 | 0.382 | 0.369 |
| Item24 | 0.090 | 0.256 | 0.336 | -0.116 | 0.200 | 0.579 |
| Item25 | 0.150 | 0.785 | -0.032 | 0.025 | 0.640 | 0.778 |
| Item26 | 0.046 | 0.343 | 0.321 | 0.397 | 0.380 | 0.550 |
| Item27 | -0.008 | 0.316 | -0.001 | 0.305 | 0.193 | 0.294 |
| Item28 | -0.067 | 0.300 | 0.069 | -0.041 | 0.101 | 0.259 |
| Item29 | -0.057 | -0.366 | 0.841 | 0.113 | 0.858 | 0.451 |
| Item30 | 0.045 | 0.043 | 0.817 | 0.056 | 0.675 | 0.590 |
| Item31 | 0.082 | -0.098 | 0.321 | 0.021 | 0.120 | 0.586 |
| Item32 | 0.094 | -0.006 | 0.498 | -0.069 | 0.262 | 0.850 |
| Item33 | -0.047 | 0.021 | 0.370 | -0.051 | 0.142 | 0.250 |
| Item34 | 0.037 | -0.317 | 0.483 | -0.381 | 0.480 | 0.545 |
| Item35 | 0.023 | 0.052 | 0.579 | -0.038 | 0.340 | 0.250 |
| Item36 | -0.216 | 0.019 | 0.486 | 0.083 | 0.290 | 0.250 |
| Item37 | 0.082 | -0.304 | 0.525 | 0.026 | 0.375 | 0.745 |
| Item38 | 0.009 | -0.071 | 0.550 | -0.048 | 0.310 | 0.250 |
| Item39 | 0.039 | -0.107 | 0.274 | -0.016 | 0.088 | 0.696 |
| Item40 | 0.131 | 0.370 | 0.592 | -0.190 | 0.540 | 0.761 |
| Item41 | -0.106 | 0.193 | 0.486 | -0.131 | 0.302 | 0.402 |
| Item42 | 0.054 | -0.017 | 0.666 | -0.045 | 0.448 | 0.565 |
| Item43 | -0.014 | 0.170 | 0.146 | -0.308 | 0.145 | 0.536 |
| Item44 | -0.102 | -0.352 | -0.068 | 0.821 | 0.812 | 0.250 |
| Item45 | 0.139 | 0.319 | -0.064 | 0.552 | 0.430 | 0.386 |
| Item46 | -0.049 | -0.044 | -0.208 | 0.807 | 0.698 | 0.250 |
| Item47 | 0.223 | 0.076 | 0.006 | 0.826 | 0.738 | 0.808 |
| Item48 | -0.059 | -0.127 | 0.391 | 0.236 | 0.228 | 0.578 |
| Item49 | -0.079 | -0.104 | 0.060 | 0.671 | 0.471 | 0.287 |
| Item50 | -0.377 | -0.070 | -0.016 | 0.707 | 0.648 | 0.254 |
| Item51 | -0.027 | -0.074 | -0.007 | 0.232 | 0.060 | 0.250 |
| Item52 | -0.241 | 0.180 | -0.048 | 0.815 | 0.757 | 0.656 |
| Item53 | -0.016 | 0.330 | -0.344 | 0.605 | 0.594 | 0.527 |
| Item54 | 0.080 | -0.391 | -0.280 | -0.383 | 0.384 | 0.256 |
| Item55 | 0.058 | 0.111 | 0.148 | 0.469 | 0.258 | 0.561 |
